# My Name Is Nobody

**DOI:** 10.3201/eid1911.AC1911

**Published:** 2013-11

**Authors:** Polyxeni Potter

**Affiliations:** Centers for Disease Control and Prevention, Atlanta, Georgia, USA

**Keywords:** art science connection, emerging infectious diseases, art and medicine, the Sappho Painter, Odysseus Escaping from the Cave of Polyphemos, prion diseases, scrapie, my name is nobody, ancient Greek pottery, about the cover

**Figure:**
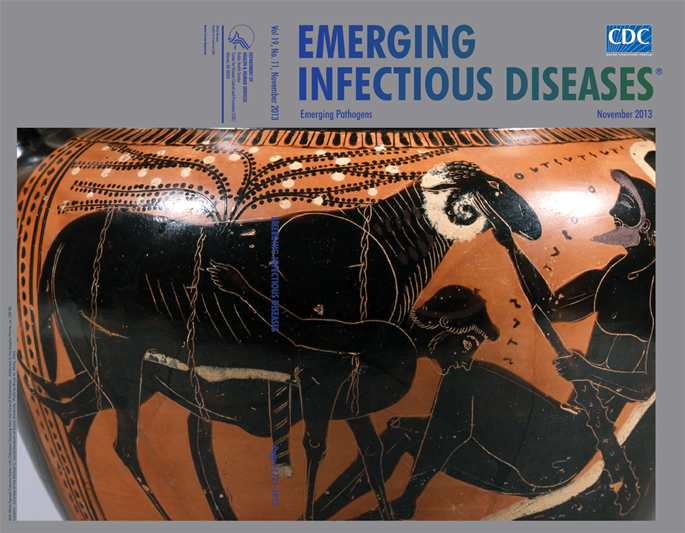
**Attributed to the Sappho Painter *Odysseus Escaping from the Cave of Polyphemos* (detail) (c. 2500 years ago) Attic black-figured column-krater, ceramic.** Courtesy of the Michael C. Carlos Museum of Emory University, Atlanta, Georgia, USA. Photo by Bruce M. White, 2004

Sweet wine, unblended, served Odysseus well in the escape with his companions from the Cyclops’ cave during his epic return to Ithaca. The wine, a gift from Maron, grandson of Dionysus, was exceptional. “When he drank it he mixed twenty parts of water to one of wine, and yet the fragrance from the mixing bowl was so exquisite that it was impossible to refrain from drinking.” Odysseus carried a large skin with this wine, in case on the way he had to deal with some unknown savage of great strength who “would respect neither right nor law.”

Mixing wine with water before drinking was a mark of civilized behavior in ancient Athens and an essential feature of the symposium, a gathering in which drinking together was intertwined with conducting business. The practice spawned a line of equipment for transporting, mixing, and consuming wine. One such implement was the krater, a vessel in which wine was diluted to the right consistency for drinking. Kraters, often too large to be used for serving, were positioned in the center of a room and sometimes were decorated with images of symposium proceedings.

Athenian pottery was common in the Mediterranean region as far back as 2,800 years ago. Clay vessels of different sizes, shapes, and uses were widely traded. And while little painting of that period has survived, even on stone, intact and fragmented painted vases from various locations abound because they were exceptionally durable, more so than metal. As a result, they became a repository, not only of painting but of religious and social norms and daily activities, from raising children to burying the dead. Historical and mythologic scenes, also used heavily, provided the opportunity to inject life scenes with life lessons.

Pottery was difficult work half art, half magic. Clay pots were created in workshops, usually led by master potters, who knew how to manipulate kiln temperature by letting air in and out at different times, never knowing for certain how the final product would turn out. Many pots were clearly signed. Others are recognizable as products of a certain workshop or area known for a distinctive style. The potter was not necessarily the painter, although many times they were one and the same. The painter who created the krater on this month’s cover was named after Sappho, the famous poet of antiquity, a popular subject for artistic representation. His name, “the Sappho painter,” came from a vase, now in a museum in Warsaw, showing perhaps the oldest portrait of Sappho, a woman playing a long-armed lyre. The name “Sappho” is incised next to the figure.

The black-figure technique, featured on the krater on this month’s cover, evolved from earlier geometric designs in Corinth and then Athens. The surface of the pot was covered with a black pigment on which details were incised that would turn red in the final stage of firing. Certain conventions prevailed in the painted scenes. Figures were flat, although not entirely without perspective. Faces were shown in profile, young men generally beardless, older women heavyset. Inscriptions floated conspicuously in-between figures.

*Odysseus Escaping from the Cave of Polyphemos* is the image on a wide mouth black-figure krater with two columnlike handles. The theme was a popular one: a famous hero set against Polyphemos—“A horrid creature, not like a human being at all, but resembling rather some crag that stands out boldly against the sky on the top of a high mountain.” The tired Odysseus and his crew found the monster’s cave. “His cheese-racks were loaded with cheeses, and he had more lambs and kids than his pens could hold.” But what he had in prosperity, Polyphemos lacked in hospitality. “The cruel wretch … gripped up two of my men at once and dashed them down upon the ground as though they had been puppies. Their brains were shed upon the ground, and the earth was wet with their blood. Then he tore them limb to limb and supped upon them.”

Homer’s hero realized that extraordinary measures would be needed for him and his companions to get out of the monster’s cave alive. “Look here, Cyclops … you’ve been eating a great deal of man’s flesh, so take this and drink some wine.” The undiluted potion had the anticipated effect. “This drinks like nectar and ambrosia all in one,” the Cyclops exclaimed. “Be so kind … as to give me some more and tell me your name at once.” Odysseus obliged. “My name is Nobody … This is what my father and mother and my friends have always called me.”

Drunk and sick, the monster fell “backwards and a deep sleep took hold upon him.” Odysseus and his men then thrust a burning beam of wood into the monster’s eye “till the boiling blood bubbled all over it as we worked it round and round.” The Cyclops cried and shouted “in a frenzy of rage and pain,” alerting his friends that Nobody was killing him, “by fraud or by force.” For Odysseus and his friends, the problem now was how to get out of the cave when Polyphemos moved the huge bolder to let the sheep out in the morning.

“The male sheep were well grown and carried a heavy black fleece, so I bound them noiselessly in threes together … there was to be a man under the middle sheep, and the two on either side.” And, “There was a ram finer than any of the others, so I caught hold of him by the back, ensconced myself in the thick wool under his belly, and flung on patiently to his fleece, face upwards, keeping a firm hold on it all the time.”

On the krater depicting the story, the faces betray no emotion. Polyphemos has just lost his vision. “Nobody,” tied with rope under the ram and clinging for dear life, awaits the outcome of his daring escapade. The ram receives a tender stroke from his master. “My good ram, what is it that makes you the last to leave my cave this morning?”

Outside the cave at last, his men safely on the ship, Odysseus cannot contain himself. “Like a craftsman, I had to leave my name on my handiwork,” he shouts. “Cyclops …. If anyone asks you who it was that put your eye out and spoiled your beauty, say it was the valiant warrior Odysseus, son of Laertes, who lives in Ithaca.”

In the times of Homer, monsters lived in caves, the mountains, and the sea. They symbolized humans’ worst fears—being eaten was one of them. “He gobbled them up like a lion in the wilderness, flesh, bones, marrow, and entrails, without leaving anything uneaten.” There was a clear dichotomy. Cyclopes were bad, sheep good. Cyclopes ate humans. Humans ate sheep and shared food and wine with others.

In our times, the landscape of monsters has expanded to include the dark side within us and even within sheep. In the absence of the mythical Cyclopes, humans can still be eaten, now by disease, which sometimes devours the brain, disabling and finally destroying the body. Unbeknown to Odysseus, his escape vessel, Polyphemos’ prized sheep, could destroy him if its self-replicating prions could be transmitted to humans. For BSE, a prion disease of domestic cattle, good evidence exists from natural exposure and laboratory studies that this could happen. For scrapie, a prion disease of sheep, the evidence is not there, although the risk remains unknown.

Like pottery making, models of disease are fraught with uncertainties. They can be influenced by subtle differences in prion strains from different species of animals. The difficulty lies in demonstrating human susceptibilities in animal models. However, animals are not humans, humans are not identical to each other, and small risk is difficult to demonstrate statistically. We cannot tell if atypical scrapie prions cause human disease because, despite the names we ascribe to atypical proteins, we do not know who they are or what they are capable of until, like Odysseus, they finally announce themselves.
